# The combination of mupirocin and *Kayvirus* broadens the decolonization effect against *Staphylococcus aureus*

**DOI:** 10.1007/s00253-026-13814-0

**Published:** 2026-04-16

**Authors:** Alena Siváková, Eliška Figallová, Tibor Botka, Lukáš Vacek, Jan Tkadlec, Jan Vrbský, Martin Osowski, Petr Petráš, Dominika Polaštík Kleknerová, Milada Dvořáčková, Pavlína Urbanová, Roman Pantůček, Filip Růžička

**Affiliations:** 1 Department of Microbiology, Faculty of Medicine, Masaryk University and St. Anne’s University Hospital, Brno, Czech Republic; 2 Department of Experimental Biology, Faculty of Science, Masaryk University, Brno, Czech Republic; 3 Department of Medical Microbiology, Second Faculty of Medicine, Charles University and University Hospital Motol and Homolka, Prague, Czech Republic; 4 National Reference Laboratory for Staphylococci, National Institute of Public Health, Prague, Czech Republic

**Keywords:** *Staphylococcus aureus*, Panton-Valentine leukocidin, Decolonization, Mupirocin, Phage therapy, *Kayvirus*

## Abstract

**Abstract:**

*Staphylococcus aureus* strains cause a wide range of infections in humans, often with the potential for complications such as surgical site infections. The production of Panton-Valentin leukocidin (PVL) by certain strains of *S. aureus* is clinically associated with chronic or recurrent infections, which typically require decolonization, most often with mupirocin. As increased mupirocin use promotes the emergence of resistance, this study investigated the coadministration of mupirocin and therapeutic *Kayvirus* bacteriophage as a potential strategy to enhance treatment efficacy and prevent the development of new resistance. We collected and evaluated 37 PVL-encoding *S. aureus* strains from wound samples. Among these, 22% were methicillin-resistant, and 11% were resistant to the tested phage, but all were susceptible to mupirocin. To assess interactions between mupirocin and the phage in PVL-positive strains with varying levels of mupirocin resistance, we used lysogenization by PVL-encoding phage and adaptive laboratory evolution of clinical strains. In mupirocin-susceptible strains, lytic phage efficacy decreased due to altered protein synthesis caused by the interaction of mupirocin with isoleucyl-tRNA synthetase, whereas mupirocin efficacy was unaffected. In contrast, the advantage of combined administration was observed in mupirocin-resistant strains susceptible to phages, as their altered or alternative synthetase allowed protein synthesis to continue, enabling phage proliferation and bacterial lysis, even in the presence of mupirocin. This in vitro study demonstrates that mupirocin in combination with *Kayvirus* broadens the spectrum of strains susceptible to treatment and that the phage used prevents the development of mupirocin resistance.

**Key points:**

• *Mupirocin-phage combination broadens the anti-staphylococcal effect in vitro.*

• *Combination treatment reduces the emergence of mupirocin resistance.*

• *Mupirocin action is not inhibited by phage therapy.*

**Supplementary Information:**

The online version contains supplementary material available at 10.1007/s00253-026-13814-0.

## Introduction

Infections remain a leading cause of death worldwide, and reducing their impact is an urgent global public health priority (Murray et al. 2022). *Staphylococcus aureus* is the predominant human pathogen, with an all-cause, age-standardized mortality rate of 14.6 per 100,000 population (Ikuta et al. 2022). It causes a wide range of infections, including bloodstream infections (BSIs) and skin and soft tissue infections (SSTIs), of which it is the leading cause (Tong et al. 2015). Approximately 30% of the human population carries this bacterium asymptomatically (Wertheim et al. 2005). However, *S. aureus* carriage can pose a significant risk in certain clinical contexts such as cardiac surgery (Langenberg et al. 2018), acute leukemia (Ghaffary et al. 2024), and hemodialysis (Hassoun-Kheir et al. 2023), requiring eradication. Due to the rise of antimicrobial resistance (AMR), methicillin-resistant *S. aureus* (MRSA) has been a major focus of recent research (Murray et al. 2022). Although MRSA decolonization has been achieved using antibiotic-based regimens, such approaches carry the risk of selecting for resistant *S. aureus* strains (Eed et al. 2019).

The clinical significance of *S. aureus* is amplified by its wide array of virulence factors, including toxins with superantigen activity and cytotoxins. Among the toxins, Panton-Valentine leukocidin (PVL), encoded by prophage-borne *lukS-PV* and *lukF-PV* genes, is a well-known toxin that causes leukocyte destruction and tissue necrosis. PVL is commonly associated with chronic necrotic skin lesions and severe necrotizing pneumonia (Genestier et al. 2005; Löffler et al. 2010). Patients colonized with PVL-producing *S. aureus* strains commonly suffer from recurring skin lesions and have an elevated risk of surgical site infections (Hoppe et al. 2019; Leistner et al. 2022); therefore, they may benefit from prophylactic decolonization.

Traditional *S. aureus* decolonization regimens utilize nasally administered mupirocin combined with chlorhexidine soap for body washing. However, this treatment fails in upwards of 30% of patients and must be repeated (Cool-Foley et al. 1991; Rossi et al. 1996; Wagenlehner et al. 2007; Ammerlaan et al. 2011). Colonization of non-nasal sites, such as the throat or wounds, is associated with higher rates of eradication failure (Mollema et al. 2010; Lindgren et al. 2018). Resistance to both chlorhexidine (CHX-R) and mupirocin (Mu-R) has been reported, with variable prevalence across regions: for example, Mu-R 9.1% and CHX-R 5.8% in Saudi Arabia, and Mu-R 19.3% in the USA (Antonov et al. 2015; Yankey and Isaacson 2018). Mupirocin acts by inhibiting bacterial isoleucyl-tRNA synthetase, thereby blocking protein synthesis. Two types of mupirocin resistance have been previously described: high-level resistance (Mu-HR), mediated by an alternative isoleucyl-tRNA synthetase encoded by the *mupA* or *mupB* genes, which are typically carried on plasmids (Hodgson et al. 1994; Seah et al. 2012), and low-level resistance (Mu-LR), resulting from *mupA* carried on the chromosome or from point mutations in the wild-type *ileS* gene encoding isoleucyl-tRNA synthetase. Although Mu-LR strains respond to mupirocin treatment, this type of resistance has been described as the cause of decolonization therapy failure (Hetem and Bonten 2013).

Novel approaches are urgently needed to enhance the efficacy of decolonization and combat the emergence of resistance. Bacteriophages offer promising therapeutic potential because of their pathogen specificity and synergy with antibiotics (Comeau et al. 2007; Obořilová et al. 2025). In this study, we investigated the combination of mupirocin with the therapeutic phage 812K1/420 of the *Kayvirus* genus (Pantůček et al. 1998; Botka et al. 2019). We evaluated, in vitro*,* the efficacy of this combined therapy against clinical PVL+ *S. aureus* isolates and assessed its potential in preventing de novo emergence of mupirocin resistance. We focused on PVL+ strains as a typical target of decolonization; however, our conclusions apply to *S. aureus* in general.

## Materials and methods

### *Staphylococcus aureus* isolates

Between January 2021 and December 2022, 17 *S. aureus* isolates positive for PVL-encoding genes (PVL+ strains) and two non-PVL strains were collected from adult patients treated for SSTIs in various hospital departments (surgery, internal medicine, psychiatry, oncology, and others) (Table S1). Samples were collected from four hospitals that provide care to patients from the Moravian region (labeled AS): St. Anne’s University Hospital, the Center for Cardiovascular Surgery and Transplantation in Brno, Masaryk Memorial Cancer Institute, and the Hospital of the Merciful Brothers in Brno, Czech Republic. Samples from suspected SSTIs (e.g., pus and wound swab) were cultured on blood agar (Oxoid, UK) and blood agar with 10% NaCl (Oxoid, UK). *S. aureus* strains were deposited in the Collection of Microorganisms of the Department of Microbiology, St. Anne’s University Hospital and Faculty of Medicine, Masaryk University, Czech Republic. The National Reference Laboratory (NRL) for Staphylococci of the National Institute of Public Health (NIPH) provided 20 PVL+ *S. aureus* isolates (labeled NRL/St) from SSTIs in hospitals across the Bohemian region (Table S1). Bacterial identification was carried out using MALDI-TOF MS (Bruker Daltonics, USA) according to the manufacturer’s instructions. All bacterial isolates were obtained from routine diagnostic samples, with only anonymized data regarding their origin included in this study.

### Detection of the Panton-Valentine leukocidin genes

The PVL *lukS-PV* and *lukF-PV* genes (GenBank accession: X72700.1) and the *S. aureus* species-confirming *nuc* gene were detected using real-time PCR and primers, as described previously (Galia et al. 2019). PVL+ *S. aureus* strain NRL/St 22/167 (isolated from a blood culture, NIPH NRL collection) and NRL/St 22/195 (Table S1) served as positive controls after the PCR amplicons were confirmed using Sanger sequencing. DNA isolation was performed using the 3DMed isolation kit (3D Biomedicine Science and Technology Co., China) and the KingFisher Flex device (Thermo Scientific, USA), according to the manufacturer’s instructions. The 10 µL reaction mixture consisted of 0.5 µL of DNA template, 0.5 µL of primers (10 µM) each, 5 µL of LightCycler 480 SYBR Green I Master (Roche, Switzerland), and 3.5 µL of nuclease-free water. End-point real-time PCR was performed using a CFX96 Real-time PCR system (Bio-Rad, USA).

### Antimicrobial susceptibility testing

Using the standardized disc (Oxoid, UK) diffusion method on Mueller-Hinton agar (MHA, Oxoid, UK), all isolates were tested against cefoxitin, erythromycin, clindamycin, trimethoprim/sulfamethoxazole, tetracycline, mupirocin, gentamicin, rifampicin, linezolid, ciprofloxacin, tigecycline, and ceftaroline. *S. aureus* ATCC 25923 was used as a control strain. The minimum inhibitory concentrations (MICs) of vancomycin (Erba Lachema, Czechia) and mupirocin (Fagron a.s., Czechia) were determined in accordance with the ISO standard 20776-1:2019 using the broth microdilution method in cation-adjusted MHB (Oxoid, UK). Methicillin resistance was assayed by PCR detection of the *mecA* gene, as described by Belguesmia et al. (2021). Methicillin-resistant *S. aureus* ATCC 43300 was used as a control. For mupirocin-resistant *S. aureus* strains, resistance was verified using the microdilution method, which determined the exact MIC. All results were interpreted according to EUCAST guidelines (version 16.0, 2026). Methicillin-resistant *S. aureus* ATCC 43300 was used as a control. The mechanism of resistance to mupirocin was determined by PCR detection of *mupA,* as described by Eed et al. (2019).

### Phage 812K1/420 susceptibility testing

Phage strains 812 (wild type) and 812K1/420 have been described previously (Pantůček et al. 1998; Botka et al. 2019). The ability of the phage to propagate on the analyzed strains was confirmed by the appearance of single plaques after a drop test using serial dilution of the phage lysate as previously described by Pantůček et al. (1998). Next, to demonstrate the ability of phage 812K1/420 to overcome the abortive system determined by Pdp_Sau_ protein, we tested the phage susceptibility of the *S. aureus* strains RN4220, RN4220 (53^+^), NCTC 8325, and NRL 02/947 (Kuntová et al. 2023).

### Determination of clonal relatedness of Panton-Valentine leukocidin-producing *S. aureus* strains

PVL+ *S. aureus* isolates were characterized by *spa* typing as described by Harmsen et al. (2003). Individual *spa* types were assigned using the Ridom StaphType software package, version 2.2.1 (Ridom, Germany). Based on a previously described high concordance (0.954–0.979) between *spa-*typing and MLST in determining the clonal complex (CC) (O’Hara et al. 2016), the CCs were inferred from *spa* types using information available in the SpaServer database (http://spaserver.ridom.de), from previously published studies (Blanco et al. 2011; Egyir et al. 2014; Kinross et al. 2017; Durand et al. 2018; Klein et al. 2020; Monecke et al. 2021; Tabaja et al. 2021; Rakonjac et al. 2022; Quero et al. 2023; Yin et al. 2024), or from whole-genome sequences using MLST 2.0 tool (Larsen et al. 2012).

Pulsed-field gel electrophoresis (PFGE) was conducted as described by Pantůček et al. (1996). *Sma*I (New England Biolabs, USA) macrorestriction fragments were separated with a CHEF Mapper XA System (Bio-Rad Laboratories, CA) using 1.2% agarose in 1 × Tris-acetate-EDTA (TAE) buffer at 14 °C, 5.5 V/cm, pulse times of 1–55 s for 24 h. *Sma*I-cleaved DNA from *S. aureus* strain NCTC 8325 served as the ladder. The gel was stained with ethidium bromide for 30 min, destained in water, and visualized under UV light at a wavelength of 302 nm. PFGE analysis was performed using the BIONUMERICS software version 8.1 (bioMérieux, Applied Maths, Belgium).

### Preparation of PVL+ strain with high-level resistance to mupirocin

To prepare the PVL+ Mu-HR strain, a non-PVL Mu-HR SSTI isolate AS 21/64 (Table S1) was lysogenized (Holochová et al. 2010) using the PVL-converting temperate phage phiSa2 07/415, which originated from the MRSA strain NRL/St 07/415. Next, single colonies were cultivated three times in medium containing 1 mM sodium citrate to obtain a pure lysogen culture. PCR detection of the prophage and PVL genes, as well as PFGE of *Sma*I macrorestriction fragments, confirmed the presence of an integrated prophage (Fig. S1). One culture was randomly selected, and the strain was designated AS 21/64L.

### Preparation of PVL+ strains with low-level resistance to mupirocin

Two PVL+ Mu-LR strains were prepared using adaptive laboratory evolution (ALE). PVL+ strains NRL/St 21/350 and AS 22/149 were repeatedly exposed to mupirocin to evolve low-level resistance, which is described as an MIC range of 8–64 mg/L (Patel et al. 2009). ALE was performed in a 96-well plate using a liquid culture of approximately 1 × 10^6^ CFU/mL in Mueller-Hinton broth (MHB; Oxoid, UK) in three biological replicates. The concentration of mupirocin was initially set at the MIC for each strain. Every 24 h, it was doubled, and 100 µL of the culture was transferred into a new well with fresh medium. The remaining 100 µL of the culture was plated on Mueller-Hinton agar (MHA) at the respective mupirocin concentrations and incubated overnight at 37 °C. Individual colonies were then picked and streaked onto MHA with or without mupirocin as a growth control. Strains designated NRL/St 21/350R and AS 22/149R were cultivated from randomly selected resistant colonies on plates with 8 mg/L mupirocin.

### Measurement of the combined effect of mupirocin with phage on distantly related strains

To determine the combined effect of administration, PVL+ isolates representing distinct PFGE profile groups: NRL/St 21/219, AS 22/130, NRL/St 20/786, NRL/St 21/377, NRL/St 21/350, AS 22/149 (selected randomly), and AS 21/64L were used. The strains were grown overnight on separate plates with 1.5% Tryptone Soya Agar (TSA; Oxoid, UK), and individual colonies were inoculated into 10 mL of MHB with adjusted Ca^2+^ and Mg^2+^ ions according to CLSI standards. Fresh cultures were grown at 37 °C with orbital shaking (120 rpm) until the late exponential phase and then diluted to 2 × 10^8^ CFU/mL. In addition to the growth and negative controls, seven conditions of phage and/or mupirocin (0.03 mg/L) treatment were tested in a 96-well plate (JetBiofil, China): (1) phage immediate addition, (2) phage addition after 8 h, (3) immediate addition of mupirocin, (4) mupirocin addition after 8 h, (5) addition of mupirocin immediately and phage after 8 h, (6) addition of phage immediately and mupirocin after 8 h, and (7) simultaneous immediate addition of phage with mupirocin. The input ratio (IR or MOI_INPUT_) of phages to bacteria (PFU/CFU) was 0.1. Measurements were performed over 48 h at 37 °C with mild shaking in 10-min intervals, using a spectrophotometer (Tecan Infinite M200 PRO, Tecan Trading AG, Switzerland). Optical density (OD) was measured at 850 nm, the optimal wavelength for evaluating interactions between bacterial cultures and antibiotics (Truong et al. 2021). A viable cell count of the remaining bacteria was performed after 48 h of incubation by plating the cells, resuspended in an equal volume of fresh medium adjusted with citrate, on 1.5% TSA. All experiments were performed in biological triplicates and technical duplicates.

### Spectrophotometric measurement of the effect of mupirocin and phage on Mu-HR PVL+ strain

Strain AS 21/64L was grown overnight on 1.5% TSA, and individual colonies were inoculated into 10 mL of MHB with adjusted Ca^2+^ and Mg^2+^ ions according to CLSI standards. Fresh cultures were grown at 37 °C with orbital shaking (120 rpm) until the late exponential phase and then diluted to 2 × 10^8^ CFU/mL. In addition to growth and negative control, the following treatments were tested in a 96-well plate: mupirocin at 0.03, 4, and 128 mg/L; phage (IR 0.1); and phage with mupirocin at all three concentrations. Agents were immediately added to the bacterial culture in plates. Measurements (OD_850_) were performed over 24 h at 37 °C with mild shaking in 10-min intervals, using an Infinite M200 PRO microplate reader. The experiment was performed in nine biological replicates.

### Measurement of the combined effect of mupirocin with phage on strains with various susceptibility profiles

Representative *S. aureus* PVL+ strains with different susceptibility to mupirocin and the phage were selected to determine the efficacy of the combined administration of mupirocin and the phage, that is, a phage-susceptible and mupirocin-susceptible strain NRL/St 21/350, a phage-resistant and mupirocin-susceptible strain NRL/St 20/303, a phage-susceptible and mupirocin-resistant strain with a low level of resistance (Mu-LR) NRL/St 21/350R, and a phage-susceptible and mupirocin-resistant strain with a high level of resistance (Mu-HR) AS 21/64L. A non-PVL strain AS 22/143 served as a double-resistant control. 96-well cell culture plates were inoculated with representative *S. aureus* strains (2 × 10^8^ CFU/mL) in MHB with adjusted Ca^2+^ and Mg^2+^ according to CLSI standards, phage 812K1/420 (IR = 0.1), and mupirocin at final concentrations of 4 mg/L and 128 mg/L. Microtiter plates were incubated at 37 °C for 24 h with a 10-s shaking interval for each 10-min measurement period in a Tecan Infinite M200 PRO microplate reader, with OD measured at 850 nm. After incubation, the plate was centrifuged at 500 × g for 10 min to pellet bacterial debris and cells. The growth medium containing the phage/antibiotic was removed, and the cells were resuspended in an equal volume of fresh medium with citrate adjustment. Viable cell counts were determined using the plating method. Measurements were performed for growth control, growth with the addition of mupirocin alone, growth with the addition of phage alone, and growth with a combination of phage and mupirocin in biological triplicates and technical duplicates.

### Whole-genome sequencing, assembly, and bioinformatics

The DNA of wild-type strains and their Mu-LR derivatives was isolated as previously described (Fišarová et al. 2021), with a modification in the final step: DNA was extracted from the supernatant using a Genomic DNA Clean & Concentrator-25 kit (ZymoResearch, USA) according to the manufacturer’s instructions.

For sequencing on the Oxford Nanopore Technology platform, libraries were prepared using a Ligation Sequencing Kit V14 (SQK-LSK114) according to the manufacturer’s instructions and sequenced using FLO-FLG114 flow cells (R10.4.1) in a MinION device (Oxford Nanopore Technologies, UK). The device was controlled using MinKNOW version 24.02.16 (Oxford Nanopore Technologies, UK), which was also used for base-calling (super accuracy mode) and trimming.

Complete genomes were assembled using Flye version 2.9.5 (Lin et al. 2016) and annotated using the NCBI Prokaryotic Genome Annotation Pipeline (Li et al. 2021). Resistance genes were identified using AMRFinderPlus version 3.12.8 (Feldgarden et al. 2021) with the V3.12–2024-05-02.2.2 database using a species-specific point mutation search set for *S. aureus*. Whole-genome sequences were aligned using the Mauve Plugin (Darling et al. 2004), and multiple sequence alignment was performed using Clustal Omega version 1.2.3, both in Geneious Prime version 2025.2.2 (https://www.geneious.com). Changes in isoleucyl-tRNA synthetase (IleS) amino acid sequences were compared to the NCBI reference sequence WP_000384706.

### Phage-mupirocin interaction assessment

Viable cell count (CFU/ml) data were converted to percentages, with 100% assigned to the highest observed value. These data were arranged according to the SynergyFinder v3.18.0 manual (Ianevski et al. 2022) and processed in R v4.5.2 with the additional synergyfinder v3.18.0 package (Zheng et al. 2022), which was also used for descriptive statistics. For data processing, the input data were transformed and processed using a built-in “viability” option. The phage-mupirocin interaction was calculated using Bliss and Loewe additivity models.

### Statistical analysis

For each *S. aureus* strain, differences in bacterial viable cell counts (CFU/mL) among treatment groups were assessed using Kruskal-Wallis followed by Dunn’s multiple comparison post hoc test with Benjamini-Hochberg false discovery rate correction in R (version 4.5.2). Differences in final blank-corrected OD_850_ values among treatment groups were determined using one-way ANOVA followed by Tukey’s honest significant difference (HSD) test in R. Statistical significance was set at *p* < 0.05. Results of the post hoc tests are presented using letter-based groupings, i.e., treatment groups sharing a common letter do not differ significantly. The efficacy of phage/mupirocin, based on spectrophotometric measurements, was analyzed using the Centroid Index with the software Centroid_Index_Calculator designed by Hosseini et al. (2024) and originally intended for quantifying phage efficacy.

## Results

### Characterization and clinical background of *Staphylococcus aureus *isolates

Characterization of individual PVL+ *S. aureus* isolates, including antibiotic and phage susceptibility and relatedness, is shown in Table 1. All PVL+ *S. aureus* strains were susceptible to mupirocin (MIC = 0.125 mg/L), and four (10.8%) were resistant to phage 812K1/420. Clinical isolates in this study comprised 21.6% MRSA and 10.8% MLSB-resistant strains. A total of 16 *spa* types and 8 CCs were identified (Table 1; Table S1). PFGE fingerprint patterns after *Sma*I cleavage clustered according to assigned CCs and revealed seven restriction profiles (Fig. S2), while CC398 samples were not cleavable as was previously described (Bens et al. 2006).

**Table 1:** Characterization of Panton-Valentine leukocidin-producing (PVL+) *S. aureus* strains under study

Strain	Resistance profile	MIC MUP (mg/L)	Phage susceptibility	PFGE pattern	*spa* type	CC
AS 21/97	-	0.125	S	IV	t355	CC152
AS 21/99	-	0.06	S	III	t021	CC30
AS 21/143	-	0.125	S	IV	t355	CC152
AS 21/152	-	0.125	S	IV	t355	CC152
AS 21/157	-	0.125	S	IV	t355	CC152
AS 22/3	-	0.125	S	IV	t355	CC152
AS 22/71	-	0.125	S	IV	t355	CC152
AS 22/96	-	0.125	S	V	t6465	CC121
AS 22/101	-	0.125	S	IV	t1172	CC152
AS 22/103	-	0.125	S	IV	t355	CC152
AS 22/105	-	0.125	S	III	t433	CC30
AS 22/107	-	0.125	S	III	t433	CC30
AS 22/112	-	0.125	S	IV	t355	CC152
AS 22/130	-	0.125	S	IV	t355	CC152
AS 22/149	-	0.125	S	VII	t3207	CC45
AS 22/150	iMLS_B_	0.125	S	III	t342	CC30
AS 22/155	-	0.125	S	IV	t355	CC152
NRL/St 20/085	-	0.125	R	III	t1848	CC30
NRL/St 20/095	-	0.125	S	III	t021	CC30
NRL/St 20/168	MRSA, MLS_B_, TE	0.125	S	NT	t021	CC30
NRL/St 20/183	-	0.125	S	III	t433	CC30
NRL/St 20/197	MRSA, ERY, CIP	0.125	S	II	t008	CC8
NRL/St 20/212	-	0.125	S	III	t021	CC30
NRL/St 20/269	MRSA, ERY, TE, CIP	0.125	R	III	t276	CC30
NRL/St 20/303	MRSA, TE, CN	0.125	R	I	t903	CC1153
NRL/St 20/382	-	0.125	S	III	t433	CC30
NRL/St 20/621	MRSA, MLS_B_, TE	0.125	S	NT	t034	CC398
NRL/St 20/632	-	0.125	S	III	t021	CC30
NRL/St 20/715	SXT	0.125	S	V	t21515	UNK
NRL/St 20/786	-	0.125	S	V	t435	CC121
NRL/St 20/905	MLS_B_	0.125	S	NT	t034	CC398
NRL/St 21/049	SXT	0.125	S	V	t7002	CC121
NRL/St 21/219	MRSA, ERY, CIP	0.125	S	II	t008	CC8
NRL/St 21/350	-	0.125	S	III	t433	CC30
NRL/St 21/377	MRSA, TE	0.125	R	VI	t044	CC80
NRL/St 22/195	MRSA, ERY, CIP	0.125	S	II	t008	CC8
NRL/St 22/197	-	0.125	S	IV	t355	CC152

*MIC MUP*, minimum inhibitory concentration of mupirocin; *MRSA*, methicillin-resistant *S. aureus*; *iMLS*_*B*_, inducible type of resistance to macrolides, lincosamides, and streptogramin B; *MLS*_*B*_, constitutive resistance to macrolides, lincosamides, and streptogramin B; *ERY*, erythromycin; *CIP*, ciprofloxacin; *TE*, tetracycline; *CN*, gentamicin; *SXT*, trimethoprim/sulfamethoxazole; *S*, phage-susceptible; *R*, phage-resistant; *NT*, non-typeable due to *Sma*I resistance; *CC*, clonal complex; *UNK*, unknown CC

### Antimicrobial effect of mupirocin and phage on distinct PVL+ strains

**Fig. 1:**
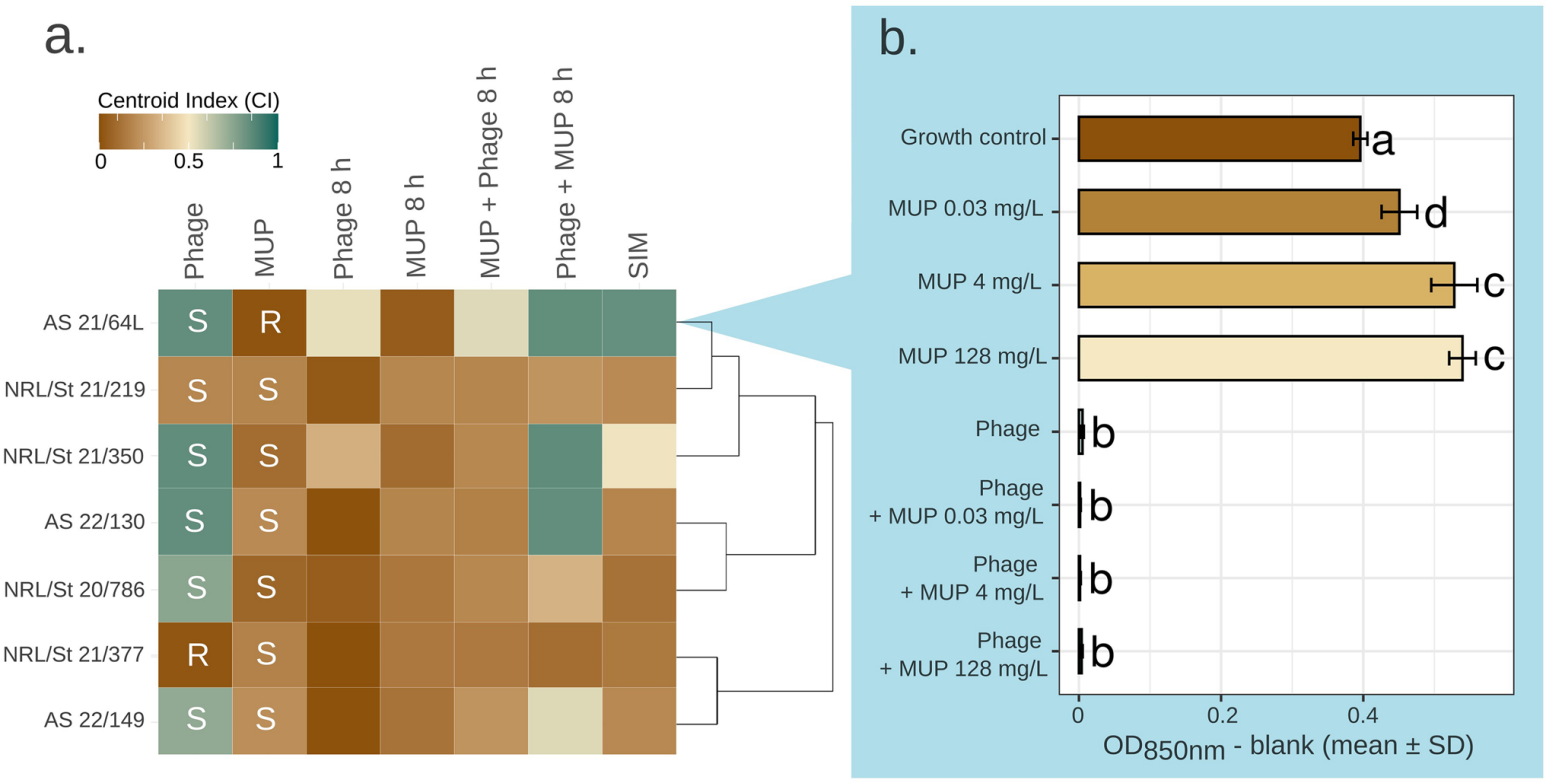
Effect of phage 812K1/420 and mupirocin (MUP) sequential and simultaneous treatment on selected PVL+ *S. aureus* isolates representing different clusters according to PFGE and *spa*-typing. **a** Heatmap showing mean Centroid Index (CI) values calculated based on 48-h spectrophotometric measurement in three biological replicates of bacterial cultures treated with phage 812K1/420 alone (“phage”), mupirocin (0.03 mg/L) alone (“MUP”), their delayed administration (“phage 8 h” and “MUP 8 h”, respectively), phage-mupirocin combination administered simultaneously (“SIM”) or sequentially (“MUP + phage 8 h”—mupirocin followed by phage after 8 h; “phage + MUP 8 h”—phage followed by MUP after 8 h). Higher CI values indicate better treatment outcomes. Strains are clustered according to PFGE analysis, without expressing their similarity as branch lengths*.* Each isolate is shown as either susceptible (S) or resistant (R) to both the phage and mupirocin. **b** Strain AS 21/64L growth reduction via optical density after 24 h of treatment using different mupirocin concentrations. Bars represent the mean ± SD of nine biological replicates. Bars not sharing a common letter (a–d) indicate significant differences (*p* < 0.05*;* ANOVA with Tukey’s HSD post hoc test). Exact *p*-values are listed in Table S3

To evaluate the potential of mupirocin therapy broadened by a therapeutic phage, seven strains, representing each PFGE group, were treated with both agents alone and in combination, administered sequentially and simultaneously (Fig. 1a)*.* For sequential administration, we chose an interval of 8 h as the time during which subpopulations resistant to the initial agent may arise, as this is often a complication of clinical treatment (Patel et al. 2009). In clinical use, mupirocin is also administered at intervals of 8 to 12 h (Poovelikunnel et al. 2015). Since phage-antibiotic synergy is usually reported for sub-inhibitory antibiotic concentrations, a ¼ MIC of mupirocin was used (0.03 mg/L).

The antibacterial effects of different treatment approaches were quantified using a Centroid Index (CI) calculated from 48-h growth measurements obtained by spectrophotometry. Changes in the position of the centroid indicate shifts in the distribution of cell population densities over time. This facilitates the comparison of bacterial growth curves, which may exhibit different trends due to various factors. The CI value ranges from 0 to 1, with 1 indicating a phage that completely eradicates the bacterial strain and 0 indicating a completely ineffective phage.

The application of phage 812K1/420 at the beginning of the measurement was highly effective in phage-susceptible strains, whereas application after 8 h had no significant eradication effect (Fig. 1a). The efficacy of mupirocin alone was generally low due to the use of a sub-inhibitory concentration, but it was noticeably higher in susceptible isolates than in the resistant strain AS 21/64L. In this strain, the addition of phage 8 h after treatment with mupirocin resulted in improved efficacy compared to mupirocin alone. Moreover, simultaneous administration led to significantly better outcomes, which was also observed in strain NRL/St 21/350.

In most cases, the efficacy of simultaneous coadministration did not differ considerably from that of mupirocin alone (Fig. 1a). In the case of mupirocin administration 8 h after phage treatment, its effect was primarily determined by whether the culture had already been eradicated due to prior phage infection. If so, the result was identical to that of treatment with phage alone; otherwise, the efficiency was reduced compared to phage administration alone. The addition of mupirocin reduced phage 812K1/420 efficacy to varying degrees in all mupirocin-susceptible strains. In contrast, the mupirocin-resistant strain AS 21/64L did not inhibit phage 812K1/420 propagation during combined treatment, even at higher mupirocin concentrations (Fig. 1b; Table S3).

Interestingly, strain NRL/St 21/350 exhibited partial phage inhibition, despite its susceptibility to mupirocin (Fig. 1a). The strain does not carry any plasmids and harbors genes and point mutations involved in fosfomycin and trimethoprim resistance (Table S2). Next, it encodes a major facilitator superfamily (MFS) efflux membrane protein, Tet38, responsible for the extrusion of organic compounds and antibiotics (Truong-Bolduc et al. 2018). We hypothesize that this may be responsible for partial mupirocin removal, allowing at least minimal phage propagation.

To evaluate the effect of the PVL-encoding prophage on susceptibility to mupirocin, strain AS 21/64L was compared with the parental strain AS 21/64 (Fig. S1). Their growth during the different treatments was very similar, except at the highest mupirocin concentration tested, where the lysogen showed better growth than the wild type, consistent with previously published results (Rendueles et al. 2023).

### Antimicrobial effect of simultaneous treatment with mupirocin and phage on PVL+ strains with different susceptibility profiles

**Fig. 2:**
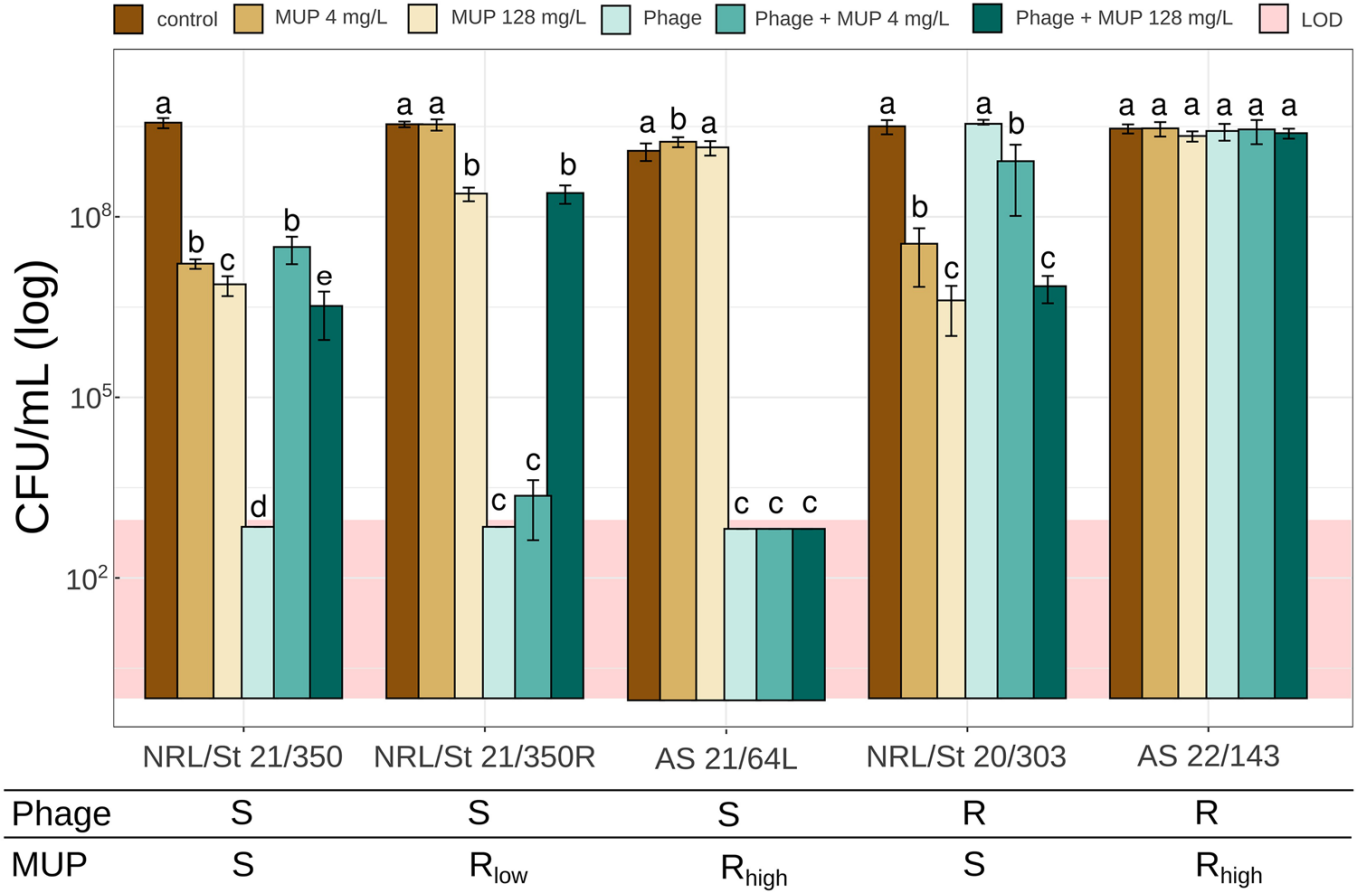
Effect of phage 812K1/420 and mupirocin (MUP) simultaneous treatment on selected PVL+ *S. aureus* isolates with different susceptibility profiles. Viable cell count after 24 h of mono- or combination treatments was assessed. Treatments included mupirocin (4 mg/L, 128 mg/L), phage alone, and phage-MUP combination. Each isolate is shown as either susceptible (S) or resistant (R) to both the phage and mupirocin. Data are shown as means ± SD of three biological replicates. Statistically distinct groups are denoted by different letters (a-e) above bars (*p* < 0.05, Kruskal-Wallis with Dunn’s post hoc test); CFU/mL values below the limit of detection (LOD) are shown in pale pink. Exact *p*-values are listed in Table S3

To assess the efficacy of the simultaneous combined treatment on strains with different phage/mupirocin susceptibility profiles, viable cell counts (CFU/mL) were determined after 24 h of treatment in five representative isolates (Fig. 2; Table S3). A sub-inhibitory concentration for Mu-LR strains of 4 mg/L (32 × MIC for susceptible strains) and a high concentration of 128 mg/L were used to assess interactions of the phage and mupirocin under different treatment conditions.

In mupirocin-susceptible isolates, the combination treatment exhibited the same efficacy as mupirocin, regardless of their susceptibility to phage (Fig. 2; Table S3). The exception was strain NRL/St 21/350, where the combination of the highest concentration of mupirocin (128 mg/L) with phage had a slightly, but statistically significantly, higher antibacterial effect than mupirocin alone. As a result, the interactions between the phage and mupirocin in this strain showed Bliss and Loewe synergy scores close to zero (additivity), while in other strains, they were strongly negative, indicating an inhibitory effect (Table S4). In the Mu-LR strain, the combination treatment with a sub-inhibitory mupirocin concentration, as well as phage alone, significantly reduced viable cell counts by at least five orders of magnitude compared to the untreated control or mupirocin-only treatment (*p* < 0.05). However, the highest concentration of mupirocin led to phage inactivation, and the outcome of combined treatment was the same as using mupirocin alone.

In the case of Mu-HR strains, the combined treatment showed the same outcome as phage treatment alone. In the phage-susceptible Mu-HR strain, treatment with both phage alone and phage-mupirocin combination resulted in significantly more effective eradication than mupirocin alone (*p* < 0.05). These results demonstrate that the addition of mupirocin does not compromise the high antibacterial effect of the therapeutic phage on Mu-HR strains. Moreover, eradication was highly effective, with no regrowth of culture after 24 h (Fig. 2), or even after 48 h (Fig. S1), as confirmed by both OD measurements and CFU/mL determination for strain AS 21/64L. Only the control non-PVL strain AS 22/143 remained unaffected due to its resistance to both agents.

### The effect of the phage-mupirocin combination on the emergence of low-level mupirocin-resistant strains

Phage reproduction relies on the bacterial protein synthesis machinery; therefore, we propose that inhibition of phage activity by mupirocin in susceptible strains is due to its interference with protein synthesis by binding to bacterial isoleucyl-tRNA synthetase (Sutherland et al. 1985). Therefore, phage propagation was maintained in strains encoding an alternative isoleucyl-tRNA synthetase (Fig. S3).

To simulate the emergence of strains with low-level resistance to mupirocin, which occurs as a result of mupirocin treatment in a clinical setting (Miller et al. 1996), strains NRL/St 21/350 (GenBank accession: JBRKZN010000001) and AS 22/149 (JBRKZM010000001) were subjected to ALE to obtain their Mu-LR derivatives. The genomes of both derived strains, designated NRL/St 21/350R (JBRKZP010000001) and AS 22/149R (JBRKZO010000001), respectively, contained de novo mutations in the *ileS* gene, which encodes isoleucyl-tRNA synthetase (Fig. S4). Because it was the only affected gene product, it can be concluded that this was the cause of the low-level mupirocin resistance.

While the amino acid sequence of NRL/St 21/350 IleS (MGS5385280) was identical to that of the reference IleS (NCBI RefSeq: WP_000384706), the homologue of AS 22/149 (MGS5377755) showed many single-nucleotide changes (Table 2; Fig. S4) and a 98.1% pairwise identity without impact on the mupirocin susceptibility of the strain. In Mu-LR strains NRL/St 21/350R (MGS5346550) and AS 22/149R (MGS5401978), three and two de novo mutations, respectively, appeared in IleS compared to the wild types. Four of them, Gly593Asp and Arg632Ile in NRL/St 21/350R and His585Tyr and Ser634Phe in AS 22/149R, were localized near the previously described Val-to-Phe mutations (V588F and V631F), which are assumed to disrupt the hydrophobic pocket in the Rossman fold responsible for mupirocin binding (Antonio et al. 2002).

**Table 2:** Amino acid substitutions in the IleS encoded by wild-type strains NRL/St 21/350 and AS 22/149 and their low-level mupirocin-resistant (Mu-LR) derivatives, compared to reference isoleucyl-tRNA synthetase (WP_000384706)

Strain	NRL/St 21/350	NRL/St 21/350R	AS 22/149	AS 22/149R
**Phenotype**	MUP-susceptible	MUP-resistant	MUP-susceptible	MUP-resistant
**aAmino acid change in IleS relative to the reference**	no	Ser191Ala*	Asn213Asp	Asn213Asp
		Gly593Asp*	Ala223Ser	Ala223Ser
		Arg632Ile*	Asn257Asp	Asn257Asp
			Glu259Gln	Glu259Gln
			Ile263Val	Ile263Val
			Asp276Gly	Asp276Gly
			Ala280Asp	Ala280Asp
			Tyr288Phe	Tyr288Phe
			Thr289Ser	Thr289Ser
			Glu306Lys	Glu306Lys
			Asp313Glu	Asp313Glu
			Gln421Lys	Gln421Lys
			Glu472Asp	Glu472Asp
			Ser570Ala	Ser570Ala
			Thr814Ser	His585Tyr*
			Asp866Glu	Ser634Phe*
			Val877Ala	Thr814Ser
				Asp866Glu
				Val877Ala

De novo mutations in Mu-LR derivatives are marked with an asterisk

**Fig. 3:**
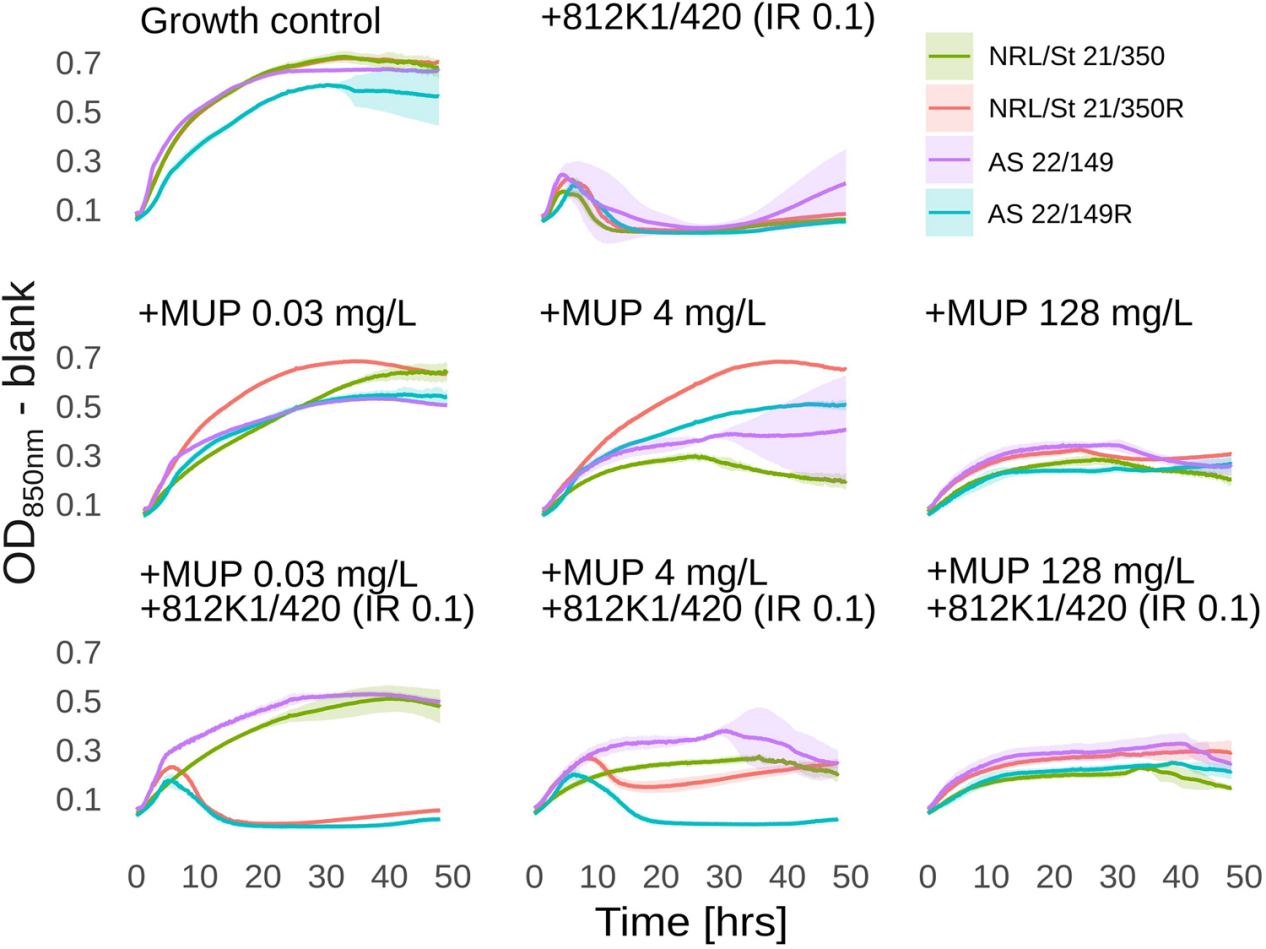
Growth properties of mupirocin-susceptible *S. aureus* strains AS 22/149, NRL/St 21/350, and their Mu-LR derivatives AS 22/149R and NRL/St 21/350R, respectively, in the presence of phage 812K1/420 (IR = 0.1), mupirocin (MUP; *c* = 0.03, 4, and 128 mg/L), and their combination. Data are shown as means ± SD of three biological replicates

To simulate changes in mupirocin concentration during treatment, three concentrations were tested (0.03 mg/L, 4 mg/L, and 128 mg/L). When low concentrations of mupirocin (0.03 mg/L and 4 mg/L) were used in combination with the phage, the inhibitory effect on Mu-LR derivatives compared to wild types was evident (Fig. 3). In contrast, when a high concentration of mupirocin (128 mg/L) was used, an almost identical inhibitory effect of the antibiotic was observed, either administered alone or in combination, and the use of phage did not provide any additional effect. Thus, when a high concentration of mupirocin was used, the growth of both the susceptible and Mu-LR strains was inhibited. However, at the lowest mupirocin concentration (0.03 mg/L), the growth of the Mu-LR strains was inhibited only in the presence of phage 812K1/420, which also contributed to the growth reduction at a concentration of 4 mg/L. Therefore, the presence of the phage could serve as protection against Mu-LR subpopulations emerging due to decreasing concentrations of mupirocin during treatment.

### Ability of phage 812K1/420 to infect Pdp_Sau_-positive strains

In some strains, the prophage-carried PVL genes can be replaced by the *pdp*_*Sau*_ abortive system gene, which targets kayviruses (Kuntová et al. 2023). Thus, selecting PVL+ isolates might overestimate the evaluation of phage lytic effect on *S. aureus*. Phage 812K1/420 was proven to propagate on Pdp_Sau_-negative prophage-less strain RN4220 as well as Pdp_Sau_-positive strains RN4220 (53^+^) and NCTC 8325, while the parental phage 812 propagated only on RN4220, demonstrating its inhibition by Pdp_Sau_ (Fig. S5). These *S. aureus* strains were chosen to observe only the prophage effect, given their shared genetic background (Herbert et al. 2010), and the results were confirmed on clinical isolate NRL 02/947 (Fig. S5).

## Discussion

Decolonization is crucial for treating and preventing *S. aureus* infections; however, conventional decolonization regimens may lead to the development of resistance to the substances used (Patel et al. 2009). Therefore, antimicrobials not applied for general treatment are typically used. Conventional decolonization regimens primarily use mupirocin, a topical antibiotic with a short biological half-life, making it unsuitable for systemic treatment (Eed et al. 2019). Despite its widespread use, clinical outcomes remain suboptimal in the long term, as strict disinfection regimens using topical antibiotics are unsuccessful in a substantial proportion of cases (Cool-Foley et al. 1991; Rossi et al. 1996; Wagenlehner et al. 2007; Ammerlaan et al. 2011). Although this study focused on PVL-positive strains, its conclusions are applicable to *S. aureus* in general.

To enhance decolonization, we evaluated the therapeutic *Kayvirus* 812K1/420, a part of the clinical phage preparation Stafal, currently registered in Slovakia but discontinued (Straka et al. 2024). Phage 812K1/420 is a derivative of phage 812a (Botka et al. 2019), which is a spontaneous mutant of phage 812 isolated on the *S. aureus* strain NCTC 8511 (53^+^) (Pantůček et al. 1998). Phage 812a can propagate on strains producing the Pdp_Sau_ protein, which induces abortive infection targeting kayviruses (Kuntová et al. 2023). This is enabled by a deletion that fuses *orf189* and *orf192* of the parental phage 812 (Botka et al. 2019). The same deletion is preserved in phage 812K1/420, and its phenotypic expression was confirmed in this study, demonstrating that targeting PVL+ *S. aureus* did not result in selection bias. Phage 812K1/420 has a broader host range also due to the deletion of the middle amidase domain of the encoded endolysin (Benešík et al. 2018; Botka et al. 2019). It previously exhibited lytic activity in 82.7% of *S. aureus* strains (Pantůček et al. 1998) and 75% of clinical MRSA isolates (Botka et al. 2019). In our study, 89.2% (*n* = 33) of 37 PVL+ isolates were phage-susceptible. Furthermore, kayviruses can be prepared in stable formulations (Komárková et al. 2025), facilitating production and distribution of the combined therapy. Combining a general-acting antibiotic with the polyvalent *Kayvirus* may provide broader efficacy than personalized single phages or phage cocktails, which are often limited by their host range (Hatfull et al. 2022). The range of strains susceptible to the phage-mupirocin combination could be further expanded by substituting with a more virulent *Kayvirus* strain or by using their cocktails (Botka et al. 2019).

Although we observed mostly an inhibitory effect of mupirocin on phage 812K1/420 in mupirocin-susceptible strains, the effect of the antibiotic itself remained unchanged. Similarly, previous studies have found that concurrent mupirocin-phage therapy accelerates MRSA eradication and decreases phage titers (Chhibber et al. 2014). Phage propagation depends on the host ability to synthesize proteins; therefore, the suppression of the phage effect results from the bacterial isoleucyl-tRNA synthetase inhibition by mupirocin. Mu-HR strains that utilize alternative tRNA ligases thus remained susceptible to phage. Next, we observed changes in the isoleucyl-tRNA synthetase gene that appeared de novo in strains that acquired a low level of resistance to mupirocin while remaining susceptible to phage.

In North America, the CC8 USA300 clone predominates in SSTIs (Talan et al. 2011). This study includes three PVL+ strains of the globally disseminated CC8, all of which were t008, MRSA, and susceptible to both phage and mupirocin. While in the USA, in 2020, 58% of invasive MRSA strains were CC8 and 2.6% were Mu-HR (Centers for Disease Control and Prevention 2025); in the Czech Republic, fewer than 5% of MRSA isolates have been assigned to CC8 (Tkadlec et al. 2021). No clinical PVL+ Mu-HR isolate was represented among the strains in this study, which could be considered a limitation. Instead, we used the non-PVL Mu-HR SSTI isolate AS 21/64 (CC1), which was lysogenized by a PVL-encoding phage. Lysogenization is a natural process, and we demonstrated that the prophage had no effect on mupirocin and phage resistance of the strain, allowing us to consider the results equivalent to those of a native PVL+ Mu-HR isolate. Although in some locations Mu-HR strains occur primarily in the most widespread CCs (Virgillio et al. 2025), plasmids encoding high-level mupirocin resistance are generally not strictly associated with any CC (Pérez-Roth et al. 2006), nor is resistance to phage 812K1/420 (Botka et al. 2019). Therefore, we assume that the effectiveness of the combined decolonization approach is not influenced by the clonal complex, but rather by strain-specific resistance mechanisms. However, the strain must be resistant to both agents to prevent the antimicrobial effect of the mupirocin-phage combination.

As mupirocin concentration decreases post-application due to its instability and metabolization (Conly and Johnston 2002; Poovelikunnel et al. 2015), sub-inhibitory concentrations may lead to selection of Mu-LR subpopulations, which were effectively suppressed by phage 812K1/420 in vitro. Thus, combination therapy may prevent the emergence of new Mu-LR subpopulations during the declining phase of antibiotic efficacy, enabling prolonged treatment, which is otherwise limited to a few days due to concerns regarding resistance development (Poovelikunnel et al. 2015). Simultaneous therapy may also prevent the emergence of resistance to either agent, as observed in clinical practice (Schooley et al. 2017). Due to the rising antibiotic resistance and limited new agents, novel applications of existing agents, including nanomupirocin and PEGylated chitosan, have been investigated (Azeez et al. 2024; Cern et al. 2024). Their use in combination with phages in semi-solid formulations, such as gels and creams, represents a promising treatment strategy.

In summary, combined treatment did not demonstrate synergistic effects, as the combination was not more effective in eradicating a given bacterial population than the individual components alone. However, coadministration of mupirocin with the 812K1/420 therapeutic phage broadens the spectrum of susceptible *S. aureus* strains, which is particularly important from the perspective of decolonization therapy in countries with high mupirocin resistance. Furthermore, the addition of bacteriophage inhibits the spread of mupirocin resistance by targeting strains that are both mupirocin-resistant and phage-susceptible, including those with de novo mutations conferring resistance to mupirocin. Importantly, phage addition does not compromise the overall antibacterial effect of mupirocin, supporting their potential use as a complementary therapeutic strategy.

## Supplementary Information

Below is the link to the electronic supplementary material.

ESM 1 (PDF 1.70 MB)

## Data Availability

The data underlying this article are available in the article, its online supplementary materials, and in subject-specific public repositories. Sequencing data of strains NRL/St 21/350 (Biosample SAMN52216007, genome sequence accession JBRKZN010000001), NRL/St 21/350R (Biosample SAMN52216392, genome sequence accession JBRKZP010000001), AS 22/149 (Biosample SAMN52215998, genome sequence accession JBRKZM010000001), and AS 22/149R (Biosample SAMN52216391, genome sequence accession JBRKZO010000001) are deposited within NCBI BioProject PRJNA1337322. Source data of the measurements presented in Fig.1, Fig. 2, Fig. 3, and Fig. S1 are available in the Zenodo repository (10.5281/zenodo.17530980).
