# Supplementary material for: The combination of mupirocin and Kayvirus broadens the decolonization effect against Staphylococcus aureus
Source: Appl Microbiol Biotechnol. 2026 Apr 16;110:157. doi: 10.1007/s00253-026-13814-0 (PMC13201327; doi:10.1007/s00253-026-13814-0)
Supplement: Supplementary file 1 [file 253_2026_13814_MOESM1_ESM.pdf]

Supplementary Figures & Tables

The combination of mupirocin and *Kayvirus* broadens the decolonization effect against *Staphylococcus aureus*

In Applied Microbiology and Biotechnology

Alena Siváková, Eliška Figallová, Tibor Botka, Lukáš Vacek, Jan Tkadlec, Jan Vrbský, Martin Osowski, Petr Petráš, Dominika Polaštík Kleknerová, Milada Dvořáčková, Pavlína Urbanová, Roman Pantůček, Filip Růžička

Corresponding author:
Tibor Botka, Department of Experimental Biology, Faculty of Science, Masaryk University, Kamenice 753/5, 625 00, CZ, e-mail: tibor.botka@mail.muni.cz

Fig. S1 Confirmation of lysogenization of *S. aureus* strain AS 21/64 by PVL-converting phage using PCR (A) and PFGE (B), and growth properties of parental strain and its lysogen in the presence of phage 812K1/420 (IR = 0.1), mupirocin (c = 0.03, 4, and 128 mg/L), and their combination (C)

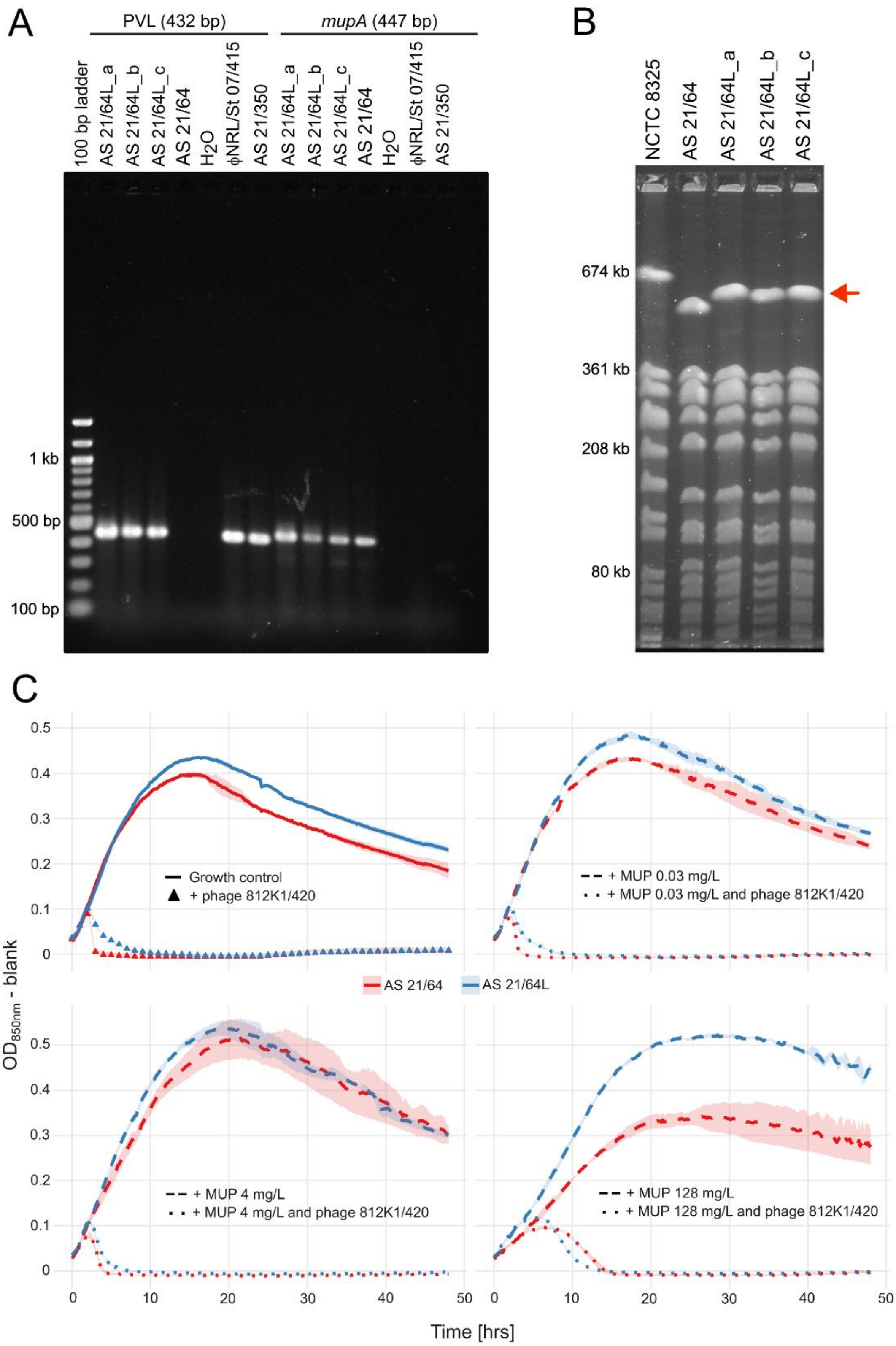

A) Lysogenic cultures were obtained from three individual colonies (a, b, c). The AS 21/64L_a strain (labeled AS 21/64L) was further used in the experiments. B) The elongation of the restriction fragment due to prophage integration is indicated by a red arrow. C) Strain cultivated in the absence of phage and mupirocin served as growth control; blank represents an OD_{850} value of the medium alone

Fig. S2 PFGE of *Sma*I-cleaved genomic DNAs of PVL+ *S. aureus* strains with a dendrogram showing the level of similarity (%) of the restriction patterns

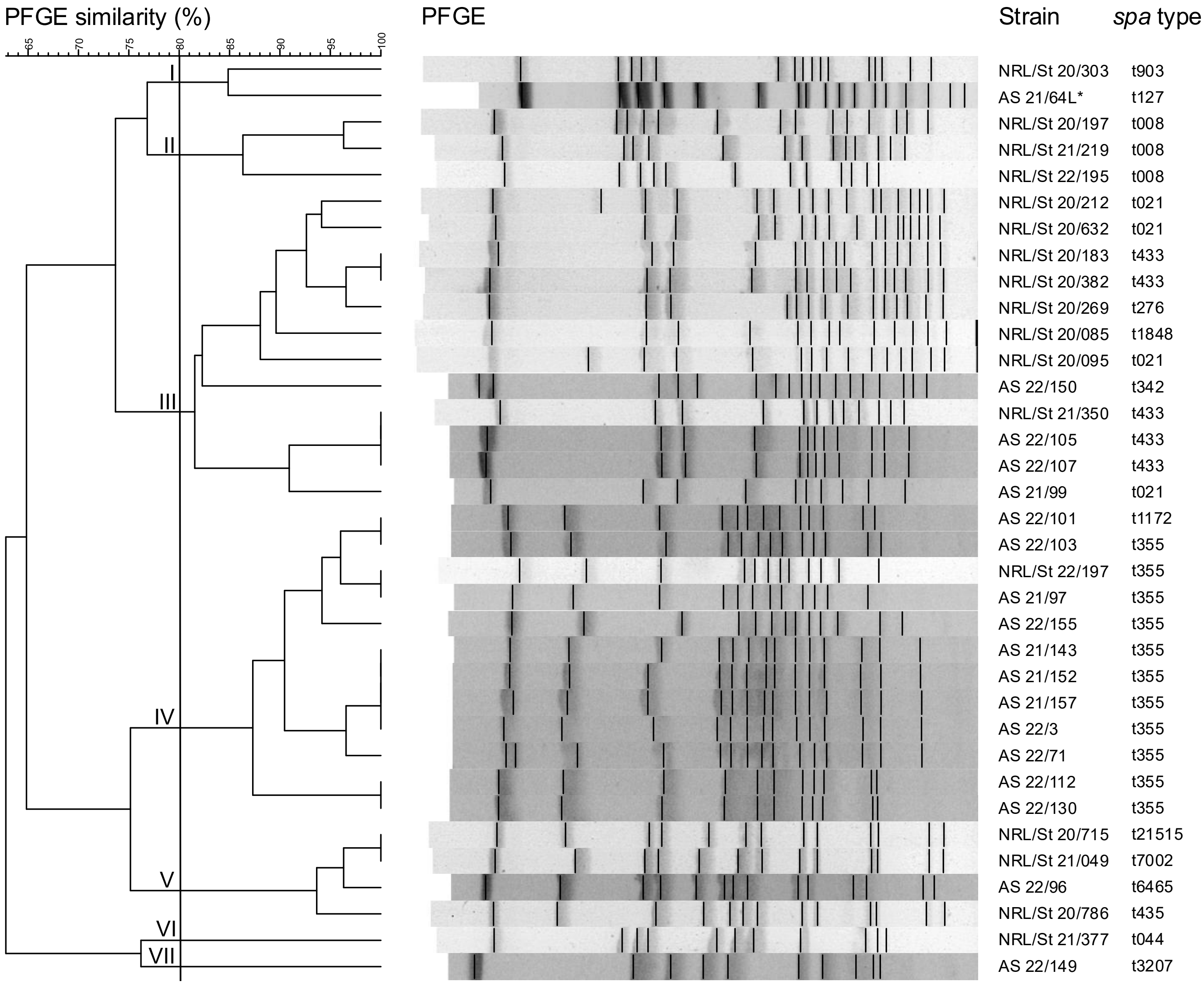

*PVL+ lysogen of Mu-HR PVL- isolate AS 21/64

Fig. S3 PCR detection of the *mupA* gene (457-bp amplicon)

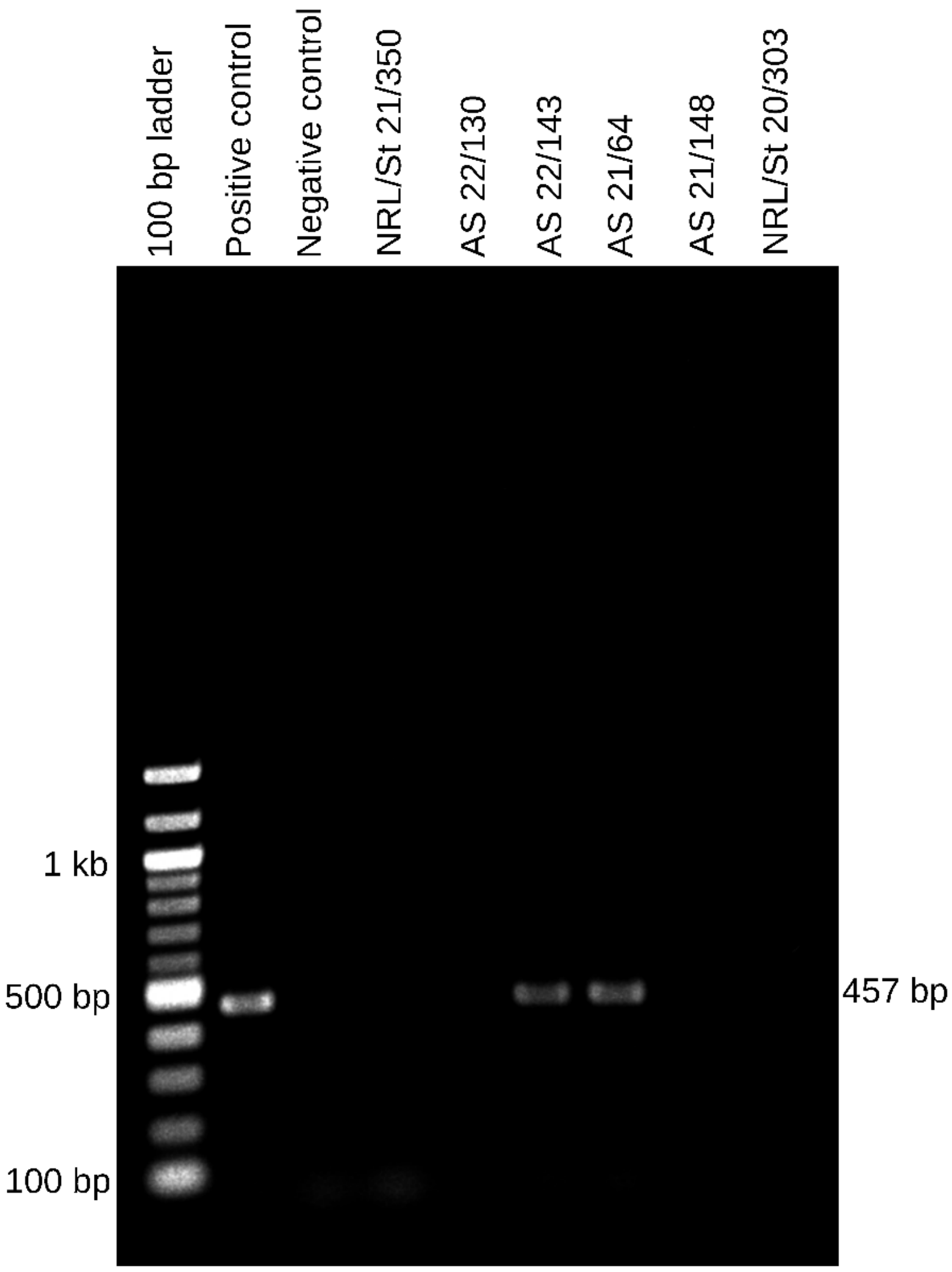

Fig. S4 Multiple alignment of reference IleS sequences with homologues of *S. aureus* strains NRL/St 21/350, AS 22/149, and their Mu-LR mutants

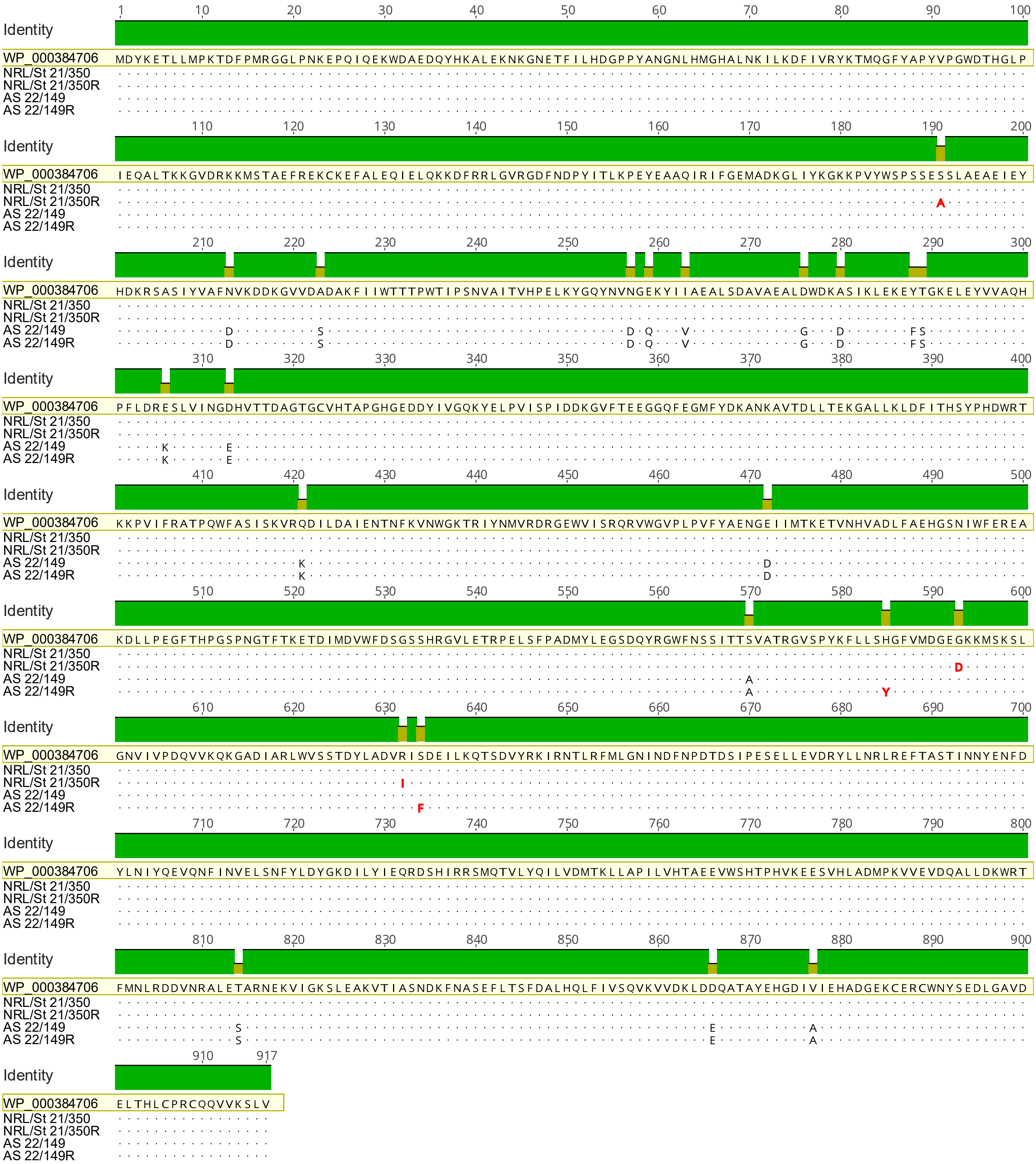

Identical positions are indicated by dots. Amino acid positions in IleS of resistant mutants that differ from parental strains are highlighted in red and bold. IleS sequence accession numbers: NRL/St 21/350 (MGS5385280), NRL/St 21/350R (MGS5346550), AS 22/149 (MGS5377755), and AS 22/149R (MGS5401978)

Fig. S5 Susceptibility of Pdp_{Sau}-negative strain RN4220 and Pdp_{Sau}-positive strains RN4220(53^{+}), NCTC 8325, and NRL 02/947 to phages 812 and 812K1/420 evaluated by drop plaque assay

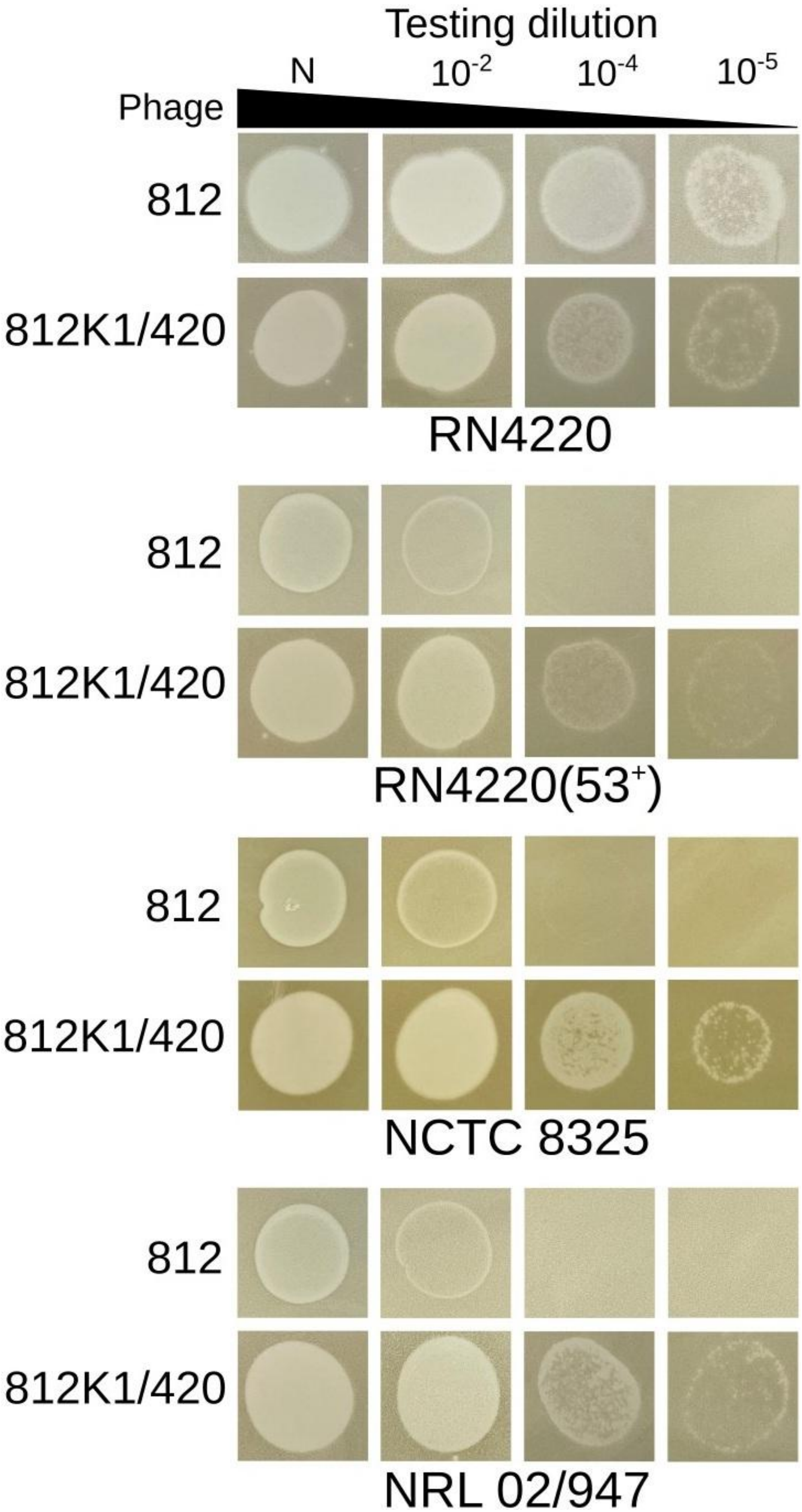

Table S1 Characterization of *S. aureus* isolates used in the study

Strain		Patient			*spa*-type	CC	Antimicrobial resistance																Phage susceptibility
									Inhibition zone diameter (mm)												MIC (mg/L)		
Laboratory	Code	Year of sampling	Gender	Age			MRSA	MLS	FOX	ERY	DA	SXT	TE	MUP	CN	RD	LZD	CIP	TGC	CPT	MIC VA	MIC MUP	
**PVL+ isolates**																							
AS	21/97	2021	F	51	t355	CC152	N	N	30	30	30	30	27	39	24	31	27	22	24	25	2	0.125	S
AS	21/99	2021	M	48	t021	CC30	N	N	29	26	28	28	27	33	31	36	31	32	30	31	2	0.06	S
AS	21/143	2021	M	68	t355	CC152	N	N	30	25	24	27	25	34	20	28	26	26	21	23	1	0.125	S
AS	21/152	2021	M	79	t355	CC152	N	N	31	29	29	34	29	34	23	34	30	28	26	29	2	0.125	S
AS	21/157	2021	M	73	t355	CC152	N	N	32	32	32	32	32	33	26	34	34	28	28	28	1	0.125	S
AS	22/3	2022	M	48	t355	CC152	N	N	27	24	24	28	24	31	30	32	32	30	26	29	2	0.125	S
AS	22/71	2022	F	65	t355	CC152	N	N	28	25	24	27	24	30	27	32	28	28	26	28	2	0.125	S
AS	22/96	2022	F	37	t6465	CC121	N	N	30	30	30	30	28	35	21	30	26	25	21	26	2	0.125	S
AS	22/101	2022	M	54	t1172	CC152	N	N	32	32	32	32	32	30	22	33	30	25	25	26	2	0.125	S
AS	22/103	2022	F	34	t355	CC152	N	N	27	25	24	28	24	32	20	28	25	24	20	27	2	0.125	S
AS	22/105	2022	M	35	t433	CC30	N	N	26	24	25	27	25	35	21	28	24	24	20	25	2	0.125	S
AS	22/107	2022	M	53	t433	CC30	N	N	28	24	24	26	24	35	20	29	25	24	20	21	2	0.125	S
AS	22/112	2022	M	51	t355	CC152	N	N	25	23	25	27	26	31	22	26	25	27	23	26	1	0.125	S
AS	22/130	2022	M	26	t355	CC152	N	N	31	26	26	31	27	31	23	32	26	26	23	27	2	0.125	S
AS	22/149	2022	M	38	t3207	CC45[†]	N	N	26	24	24	26	24	33	24	32	28	27	25	30	2	0.125	S
AS	22/150	2022	M	33	t342	CC30	N	Y	27	6	24	28	25	34	22	29	25	25	21	24	2	0.125	S
AS	22/155	2022	M	43	t355	CC152	N	N	30	32	31	31	28	33	21	30	30	27	22	26	2	0.125	S
NRL/St	20/085	2020	M	27	t1848	CC30	N	N	27	26	19	29	23	31	21	30	27	23	20	27	1	0.125	R
NRL/St	20/095	2020	F	60	t021	CC30	N	N	29	28	30	28	28	33	24	31	28	26	22	25	1	0.125	S
NRL/St	20/168	2020	F	29	t021	CC30	Y	Y	15	9	6	32	6	32	22	30	26	25	20	22	1	0.125	S
NRL/St	20/183	2020	M	9	t433	CC30	N	N	29	27	27	29	27	34	23	30	27	23	22	29	1	0.125	S
NRL/St	20/197	2020	M	34	t008	CC8	Y	N	11	6	27	29	26	31	22	31	27	6	20	20	1	0.125	S
NRL/St	20/212	2020	F	39	t021	CC30	N	N	30	28	27	32	27	34	24	30	28	25	20	30	1	0.125	S
NRL/St	20/269	2020	F	26	t276	CC30	Y	N	15	6	29	28	15	35	23	32	26	17	20	22	1	0.125	R
NRL/St	20/303	2020	M	20	t903	CC1153	Y	N	16	27	26	29	6	32	6	30	26	23	20	22	1	0.125	R
NRL/St	20/382	2020	M	92	t433	CC30	N	N	30	27	29	31	26	32	22	33	24	25	20	27	1	0.125	S
NRL/St	20/621	2020	F	15	t034	CC398	Y	Y	15	6	6	31	6	30	21	30	26	25	19	21	1	0.125	S
NRL/St	20/632	2020	M	73	t021	CC30	N	N	32	27	27	33	26	32	25	30	26	26	21	29	0.5	0.125	S
NRL/St	20/715	2020	M	43	t21515	UNK	N	N	30	27	26	19	25	30	22	30	24	23	20	28	1	0.125	S
NRL/St	20/786	2020	M	76	t435	CC121	N	N	30	26	27	30	25	32	22	30	25	27	20	27	1	0.125	S
NRL/St	20/905	2020	M	28	t034	CC398	N	Y	27	6	6	32	25	33	22	31	27	25	20	26	1	0.125	S
NRL/St	21/049	2021	F	58	t7002	CC121	N	N	27	25	26	21	24	34	22	30	25	24	19	26	1	0.125	S
NRL/St	21/219	2021	F	34	t008	CC8	Y	N	6	6	26	29	26	33	21	31	26	6	20	20	1	0.125	S
NRL/St	21/350	2021	M	17	t433	CC30	N	N	29	27	27	29	24	31	22	29	27	25	20	28	1	0.125	S
NRL/St	21/377	2021	M	7	t044	CC80	Y	N	11	27	27	30	11	33	21	31	26	22	21	23	1	0.125	R
NRL/St	22/195	2022	F	37	t008	CC8	Y	N	11	6	26	31	26	33	23	33	27	6	21	22	1	0.125	S
NRL/St	22/197	2022	M	48	t355	CC152	N	N	29	27	27	25	26	35	21	31	25	23	19	24	1	0.125	S
**non-PVL strains**																							
AS	21/64	2021	M	68	t127	CC1	N	N	30	30	28	23	33	9	6	30	30	30	24	26	1	512	S
AS	22/143	2022	M	73	t091	CC7	N	N	24	26	26	26	25	15	20	29	26	23	19	23	1	512	R

Gender: **M**ale/**F**emale

MRSA (methicillin-resistant *Staphylococcus aureus*): **Y**es/**N**o

MLS (inducibile or constitutive resistance to macrolides, lincosamides, and streptogramin B): **Y**es/**N**o

Antibiotics tested: cefoxitin (**FOX**), erythromycin (**ERY**), clindamycin (**DA**), trimethoprim-sulfamethoxazole (**SXT**), tetracycline (**TE**), mupirocin (**MUP**), gentamicin (**CN**), rifampicin (**RD**), linezolid (**LZD**), ciprofloxacin (**CIP**), tigecycline (**TGC**), and ceftaroline (**CPT**), vancomycin (**VA**), and mupirocin (**MUP**).

MIC: Minimum inhibitory concentration

Phage 812K1/420 susceptibility: **S**usceptible, **R**esistant

CC: MLST clonal complex; **UNK**: unknown CC; [†]nearest Sequence Type (ST) 45

Table S2 Determinants of antimicrobial resistance predicted in the *S. aureus* strain NRL/St 21/350

Start	Stop	Strand	Gene symbol	Sequence name	Element subtype	ATB Class	Target length	Reference sequence length	% Coverage of reference sequence	% Identity to reference sequence	Accession of closest sequence	Name of closest sequence
416875	417291	-	*fosB*	FosB/FosD family fosfomycin resistance bacillithiol transferase	AMR gene	FOSFOMYCIN	139	139	100	99.28	WP_000920239.1	FosB1/FosB3 family fosfomycin resistance bacillithiol transferase
642919	644181	+	*murA_D278E*	*Staphylococcus aureus* fosfomycin resistant MurA	point mutation	FOSFOMYCIN	421	421	100	99.29	WP_000358006.1	UDP-N-acetylglucosamine 1-carboxyvinyltransferase MurA
642919	644181	+	*murA_E291D*	*Staphylococcus aureus* fosfomycin resistant MurA	point mutation	FOSFOMYCIN	421	421	100	99.29	WP_000358006.1	UDP-N-acetylglucosamine 1-carboxyvinyltransferase MurA
1388204	1388680	+	*dfrB_L25I*	*Staphylococcus aureus* trimethoprim resistant DfrB	point mutation	TRIMETHOPRIM	159	159	100	98.11	WP_000175752.1	dihydrofolate reductase DfrB
2449415	2450770	+	*glpT_A100V*	*Staphylococcus aureus* fosfomycin resistant GlpT	point mutation	FOSFOMYCIN	452	452	100	99.56	WP_001010111.1	glycerol-3-phosphate transporter GlpT
2449415	2450770	+	*glpT_V213I*	*Staphylococcus aureus* fosfomycin resistant GlpT	point mutation	FOSFOMYCIN	452	452	100	99.56	WP_001010111.1	glycerol-3-phosphate transporter GlpT
2679261	2680610	-	*tet(38)*	tetracycline efflux MFS transporter Tet(38)	AMR gene	TETRACYCLINE	450	450	100	100	WP_001100293.1	tetracycline efflux MFS transporter Tet(38)

Resistance genes were found using AMRFinderPlus version 3.12.8 with the V3.12-2024-05-02.2 database using a species-specific point mutation search set for *S. aureus* .

Table S3 Statistical pairwise comparison of tested groups

Strain	Group 1	Group 2	Difference	Adjusted p-value	Significance
**Fig. 1b (ANOVA and Tukey's Honest Significant Difference test)**					
AS 21/64L	control	MUP 0.03 mg/L	5.50E-02	2.91E-08	***
AS 21/64L	control	MUP 128 mg/L	1.44E-01	9.54E-12	***
AS 21/64L	control	MUP 4 mg/L	1.32E-01	9.54E-12	***
AS 21/64L	control	phage	-3.91E-01	9.54E-12	***
AS 21/64L	control	phage + MUP 0.03 mg/L	-3.94E-01	9.54E-12	***
AS 21/64L	control	phage + MUP 128 mg/L	-3.92E-01	9.54E-12	***
AS 21/64L	control	phage + MUP 4 mg/L	-3.94E-01	9.54E-12	***
AS 21/64L	MUP 0.03 mg/L	MUP 128 mg/L	8.87E-02	9.57E-12	***
AS 21/64L	MUP 0.03 mg/L	MUP 4 mg/L	7.72E-02	9.87E-12	***
AS 21/64L	MUP 0.03 mg/L	phage	-4.46E-01	9.54E-12	***
AS 21/64L	MUP 0.03 mg/L	phage + MUP 0.03 mg/L	-4.49E-01	9.54E-12	***
AS 21/64L	MUP 0.03 mg/L	phage + MUP 128 mg/L	-4.47E-01	9.54E-12	***
AS 21/64L	MUP 0.03 mg/L	phage + MUP 4 mg/L	-4.49E-01	9.54E-12	***
AS 21/64L	MUP 128 mg/L	MUP 4 mg/L	-1.15E-02	8.05E-01	ns
AS 21/64L	MUP 128 mg/L	phage	-5.35E-01	9.54E-12	***
AS 21/64L	MUP 128 mg/L	phage + MUP 0.03 mg/L	-5.38E-01	9.54E-12	***
AS 21/64L	MUP 128 mg/L	phage + MUP 128 mg/L	-5.36E-01	9.54E-12	***
AS 21/64L	MUP 128 mg/L	phage + MUP 4 mg/L	-5.38E-01	9.54E-12	***
AS 21/64L	MUP 4 mg/L	phage	-5.23E-01	9.54E-12	***
AS 21/64L	MUP 4 mg/L	phage + MUP 0.03 mg/L	-5.26E-01	9.54E-12	***
AS 21/64L	MUP 4 mg/L	phage + MUP 128 mg/L	-5.24E-01	9.54E-12	***
AS 21/64L	MUP 4 mg/L	phage + MUP 4 mg/L	-5.27E-01	9.54E-12	***
AS 21/64L	phage	phage + MUP 0.03 mg/L	-3.41E-03	1.00E+00	ns
AS 21/64L	phage	phage + MUP 128 mg/L	-1.18E-03	1.00E+00	ns
AS 21/64L	phage	phage + MUP 4 mg/L	-3.57E-03	1.00E+00	ns
AS 21/64L	phage + MUP 0.03 mg/L	phage + MUP 128 mg/L	2.23E-03	1.00E+00	ns
AS 21/64L	phage + MUP 0.03 mg/L	phage + MUP 4 mg/L	-1.56E-04	1.00E+00	ns
AS 21/64L	phage + MUP 4 mg/L	phage + MUP 128 mg/L	-2.39E-03	1.00E+00	ns
**Fig. 2 (Kruskal-Wallis and Dunn's multiple comparison test with BH correction method)**					
NRL/St 20/303	control	MUP 128 mg/L	-5.05E+00	6.65E-06	***
NRL/St 20/303	control	MUP 4 mg/L	-3.39E+00	2.07E-03	**
NRL/St 20/303	control	phage	-1.41E+00	1.96E-01	ns
NRL/St 20/303	control	phage + MUP 128 mg/L	-4.61E+00	3.04E-05	***
NRL/St 20/303	control	phage + MUP 4 mg/L	-2.74E+00	1.54E-02	*
NRL/St 20/303	MUP 128 mg/L	MUP 4 mg/L	1.43E+00	1.96E-01	ns
NRL/St 20/303	MUP 128 mg/L	phage	3.89E+00	4.93E-04	***
NRL/St 20/303	MUP 128 mg/L	phage + MUP 128 mg/L	3.82E-01	7.03E-01	ns
NRL/St 20/303	MUP 128 mg/L	phage + MUP 4 mg/L	2.00E+00	8.51E-02	ns
NRL/St 20/303	MUP 4 mg/L	phage	2.24E+00	5.39E-02	ns
NRL/St 20/303	MUP 4 mg/L	phage + MUP 128 mg/L	-1.05E+00	3.38E-01	ns
NRL/St 20/303	MUP 4 mg/L	phage + MUP 4 mg/L	5.67E-01	6.11E-01	ns
NRL/St 20/303	phage	phage + MUP 128 mg/L	-3.45E+00	2.07E-03	**
NRL/St 20/303	phage	phage + MUP 4 mg/L	-1.58E+00	1.70E-01	ns
NRL/St 20/303	phage + MUP 128 mg/L	phage + MUP 4 mg/L	1.62E+00	1.70E-01	ns
NRL/St 21/350R	control	MUP 128 mg/L	-1.94E+00	7.80E-02	ns
NRL/St 21/350R	control	MUP 4 mg/L	8.27E-02	1.00E+00	ns
NRL/St 21/350R	control	phage	-4.26E+00	1.54E-04	***
NRL/St 21/350R	control	phage + MUP 128 mg/L	-1.94E+00	7.80E-02	ns
NRL/St 21/350R	control	phage + MUP 4 mg/L	-3.60E+00	1.21E-03	**
NRL/St 21/350R	MUP 128 mg/L	MUP 4 mg/L	2.03E+00	7.80E-02	ns
NRL/St 21/350R	MUP 128 mg/L	phage	-2.32E+00	5.15E-02	ns
NRL/St 21/350R	MUP 128 mg/L	phage + MUP 128 mg/L	0.00E+00	1.00E+00	ns
NRL/St 21/350R	MUP 128 mg/L	phage + MUP 4 mg/L	-1.65E+00	1.23E-01	ns
NRL/St 21/350R	MUP 4 mg/L	phage	-4.34E+00	1.54E-04	***
NRL/St 21/350R	MUP 4 mg/L	phage + MUP 128 mg/L	-2.03E+00	7.80E-02	ns
NRL/St 21/350R	MUP 4 mg/L	phage + MUP 4 mg/L	-3.68E+00	1.17E-03	**
NRL/St 21/350R	phage	phage + MUP 128 mg/L	2.32E+00	5.15E-02	ns
NRL/St 21/350R	phage	phage + MUP 4 mg/L	6.62E-01	5.86E-01	ns
NRL/St 21/350R	phage + MUP 128 mg/L	phage + MUP 4 mg/L	-1.65E+00	1.23E-01	ns
NRL/St 21/350	control	MUP 128 mg/L	-3.06E+00	6.57E-03	**
NRL/St 21/350	control	MUP 4 mg/L	-1.72E+00	1.29E-01	ns
NRL/St 21/350	control	phage	-4.95E+00	1.14E-05	***
NRL/St 21/350	control	phage + MUP 128 mg/L	-3.85E+00	8.99E-04	***
NRL/St 21/350	control	phage + MUP 4 mg/L	-1.26E+00	2.58E-01	ns
NRL/St 21/350	MUP 128 mg/L	MUP 4 mg/L	1.35E+00	2.43E-01	ns
NRL/St 21/350	MUP 128 mg/L	phage	-1.88E+00	1.12E-01	ns
NRL/St 21/350	MUP 128 mg/L	phage + MUP 128 mg/L	-7.83E-01	4.65E-01	ns
NRL/St 21/350	MUP 128 mg/L	phage + MUP 4 mg/L	1.80E+00	1.20E-01	ns
NRL/St 21/350	MUP 4 mg/L	phage	-3.23E+00	4.67E-03	**
NRL/St 21/350	MUP 4 mg/L	phage + MUP 128 mg/L	-2.13E+00	7.12E-02	ns
NRL/St 21/350	MUP 4 mg/L	phage + MUP 4 mg/L	4.53E-01	6.50E-01	ns
NRL/St 21/350	phage	phage + MUP 128 mg/L	1.10E+00	3.14E-01	ns
NRL/St 21/350	phage	phage + MUP 4 mg/L	3.68E+00	1.16E-03	**
NRL/St 21/350	phage + MUP 128 mg/L	phage + MUP 4 mg/L	2.58E+00	2.45E-02	*
AS 21/64L	control	MUP 128 mg/L	6.20E-01	6.69E-01	ns
AS 21/64L	control	MUP 4 mg/L	1.97E+00	7.25E-02	ns
AS 21/64L	control	phage	-4.67E+00	5.11E-06	***
AS 21/64L	control	phage + MUP 128 mg/L	-4.67E+00	5.11E-06	***
AS 21/64L	control	phage + MUP 4 mg/L	-4.67E+00	5.11E-06	***
AS 21/64L	MUP 128 mg/L	MUP 4 mg/L	1.35E+00	2.39E-01	ns
AS 21/64L	MUP 128 mg/L	phage	-5.29E+00	3.12E-07	***
AS 21/64L	MUP 128 mg/L	phage + MUP 128 mg/L	-5.29E+00	3.12E-07	***
AS 21/64L	MUP 128 mg/L	phage + MUP 4 mg/L	-5.29E+00	3.12E-07	***
AS 21/64L	MUP 4 mg/L	phage	-6.64E+00	1.56E-10	***
AS 21/64L	MUP 4 mg/L	phage + MUP 128 mg/L	-6.64E+00	1.56E-10	***
AS 21/64L	MUP 4 mg/L	phage + MUP 4 mg/L	-6.64E+00	1.56E-10	***
AS 21/64L	phage	phage + MUP 128 mg/L	0.00E+00	1.00E+00	ns
AS 21/64L	phage	phage + MUP 4 mg/L	0.00E+00	1.00E+00	ns
AS 21/64L	phage + MUP 128 mg/L	phage + MUP 4 mg/L	0.00E+00	1.00E+00	ns
AS 22/143	control	MUP 128 mg/L	-2.44E+00	2.21E-01	ns
AS 22/143	control	MUP 4 mg/L	-2.92E-01	8.89E-01	ns
AS 22/143	control	phage	-1.11E+00	5.01E-01	ns
AS 22/143	control	phage + MUP 128 mg/L	-1.65E+00	3.70E-01	ns
AS 22/143	control	phage + MUP 4 mg/L	-5.37E-02	9.57E-01	ns
AS 22/143	MUP 128 mg/L	MUP 4 mg/L	1.86E+00	3.15E-01	ns
AS 22/143	MUP 128 mg/L	phage	1.53E+00	3.76E-01	ns
AS 22/143	MUP 128 mg/L	phage + MUP 128 mg/L	6.82E-01	6.74E-01	ns
AS 22/143	MUP 128 mg/L	phage + MUP 4 mg/L	2.07E+00	2.91E-01	ns
AS 22/143	MUP 4 mg/L	phage	-6.14E-01	6.74E-01	ns
AS 22/143	MUP 4 mg/L	phage + MUP 128 mg/L	-1.18E+00	5.01E-01	ns
AS 22/143	MUP 4 mg/L	phage + MUP 4 mg/L	2.07E-01	8.96E-01	ns
AS 22/143	phage	phage + MUP 128 mg/L	-7.45E-01	6.74E-01	ns
AS 22/143	phage	phage + MUP 4 mg/L	8.53E-01	6.56E-01	ns
AS 22/143	phage + MUP 128 mg/L	phage + MUP 4 mg/L	1.38E+00	4.16E-01	ns

Significance: ns (p-value > 0.05), * (p-value < 0.05), ** (p-value < 0.01), *** (p-value < 0.001)

Table S4 Phage 812K1/420-mupirocin synergy assessment

Strain	MUP c (mg/L)	Synergy score	
		Loewe	Bliss
AS 21/64L	4	-94.33333783	-97.70478603
AS 21/64L	128	-93.90584927	-91.53124938
AS 22/143	4	-5.953248029	-18.08323849
AS 22/143	128	-6.575232134	-16.58602537
NRL/St 20/303	4	-71.45027646	-71.44367878
NRL/St 20/303	128	-99.31624274	-99.30514911
NRL/St 21/350	4	0.02575783	0.43693736
NRL/St 21/350	128	-0.773549501	-0.116342369
NRL/St 21/350R	4	-7.55966421	-91.11018045
NRL/St 21/350R	128	-0.383858768	0.122778251
